# Study on the Development and Structure of the Human Teeth

**Published:** 1859-04

**Authors:** Emile Magitot

**Affiliations:** Docteun en Médecine de la Faculté de Paris, ancien Eléve des Hopitaux et de l'Ecole Pratique.


### AMERICAN JOURNAL

OF

### DENTAL SCIENCE.

Vol. IX. NEW SERIES—APRIL, 1859. No. 2

### ORIGINAL COMMUNICATIONS.

### ARTICLE I.

*Study on the Development and Structure of the Human Teeth.*—By Emile Magitot, Docteun en Médecine de la Faculté de Paris, ancien Elève des Hopitaux et de l'Ecole Pratique. Translated for the American Journal of Dental Science.

#### INTRODUCTORY.

I have undertaken this work with the intention of elucidating some points which are still obscure, concerning the history of the teeth, although the number of works at present published on the development and structure of these organs is considerable. Notwithstanding these numerous works, the anatomists are far from understanding this subject. Struck by the disagreement, often absolute, on questions of fact, I have undertaken to verify and compare their assertions. I have first studied the works which recommend themselves by their high philosophical character or imposing names, such as those of Hunter, Cuvier, Geoffroy St. Hilaire, De Blainville, etc. But I have passed over some works of remote date, which have no value on account of the little influence they have exerted on science. In England and Germany I have found the greatest number of productions on odontolgeny and the structure of the teeth; prepared by all these investigations the histologists of these two countries have recently arrived at important results. But I regret to say, that I found in France only a small number of works, and, unfortunately, the most of them are governed by the influences of ancient doctrines, and betray a very incomplete acquaintance with results obtained in foreign countries and the means of investigation which have done so much to advance modern histology. The question, therefore, remains to be treated by us.

There will be found in this treatise, besides the data furnished by the works of Richard Owen, Nasmyth, J. Muller, Purkinje, Schwann, Kolliker, etc., theories which are the result of my own personal observation. This part of my work has an original aspect, for which I here take the responsibility. But I should observe that I commenced and continued these researches free from any preconceived idea of the structure and development of the teeth. It is from the daily observation of a great number of micrographic preparations that I have been conducted to new results, which I will formularize further on, and which embrace a number of points relating to science. Finally, in order to give my thoughts a nearly dogmatic form, I have offered a new theory of the formation of the teeth; this theory, already suspected by Raschkow, had not been sufficiently digested.

I will divide my subject into three parts. The *first* comprehends the study of the dental follicle in all the parts that compose it, and considered before the commencement of the production of the hard substances of the tooth. It is divided into two chapters; the first treats of the development of this follicle, and the second of its structure. The *second* part comprises the exposition of the phenomena of the development of the dental substances properly so-called, i.e. ivory, enamel and cement, in the follicle already studied.

The *third* treats of the structure of the adult tooth, i.e. the definitive disposition of the elements the evolution of which we have followed.

In adopting the order and the divisions I have indicated, I have examined the component tissues of the teeth, under three different and successive aspects.

1st. During the embryonic period, from the formation of the bulb.

2d. In the active period of development, when the dental elements are formed and disposed of.

3d. In the adult period, when the tooth is entirely complete.

Such is the method we have followed in the study of the ivory and the enamel. As to the cement, the development of which only takes place in man at the time the roots are formed, that is to say, after birth, we have not been able to find in the follicle the organ destined ultimately to produce it; nor will we be found to mention the *germ* of the *cement*, of which a praiseworthy anatomist has admitted the existence. With the pachyderms, on the contrary, the cement develops itself in the follicle of the embryo in the centre of a special organ, forming later a bony bed around the tooth; it comes out of the jaw with the tooth at the time of its eruption, and gradually wears off by the efforts of mastication, finally disappearing. But with man, where it is represented by a thin layer, hardly appreciable to the naked eye, the study of its development presents great difficulties. We have thought, therefore, to address ourselves first to animals, before directing our researches to the human follicle, but the insufficiency of material that we have had to examine, and the great extent of this study induces us to speak on this subject with the greatest reserve. Neverthelesss, we will present some considerations taken from the histology of the horse, which we believe capable of throwing some light on the question of the development of the cement. In the meantime we will investigate this point with great care.

All the descriptions of anatomical or physiological facts which this work contains have been drawn (except some parts which we have not been able to complete,) from microscopic observation of the organs and the phenomena of which they are the seat, and, for the purpose of rendering these descriptions more perfect, we have added two plates, composed of original drawings, drawn from nature, from the microscopic preparations taken from our own particular collection, or from that of our master, M. Robin, who was not only willing to put at our disposal some favorite pieces, but even made the drawings himself.

The figures 4, 5 and 6 of plate 1 and fig. 1, in plate No. 2, I owe to his extreme kindness, and for which I sincerely thank him.

### PART FIRST.

### *Study of the Dental Follicle before the Commencement of Dentification.*

#### CHAPTER I.—*Development of the Dental Follicle.*

It is not our intention to dilate on the mode of evolution of the dental follicle ; this has been done by Goodsir,* and authors have adopted and reproduced his descriptions. The time and the difficulties of such a study, which requires the examination of a great number of embryos, have not permitted us to verify the assertions of the English professor, we will limit ourselves, therefore, to a review after him, of the principal facts of this history, reserving for another time and another work, the complete study of this interesting question.

The dental follicles are formed at the expense of the buccal mucous membrane. About the middle of the tenth

* Edinburg Medical and Surgical Journal, 1838, No. 31.

week of fœtal life, a groove appears on the alveolar border of the two jaws, at the bottom of which, twenty small spheroidal protuberances are found, called dental papillæ, and destined for the formation of the milk teeth. Towards the third month, the mucous membrane, which surrounds each of the papillæ, folds itself over this organ by a mechanism, denominated by Goodsir, *involution*, and which results in the formation of an envelop, a complete sac for the papillæ, which then occupy the lowest part of it. This envelop is the dental sac. This sac, a true fold of mucus, during a certain time, communicates by the portion connected with the gum, with the buccal cavity, by means of apertures already described by Hirissant, afterwards denied by him, and which the works of Arnold, Linderer and Goodsir, seem, at last, to have demonstrated.

Before the complete occlusion of the follicular cavity a very small cavity is formed above each follicle, a kind of dental sac of reserve, intended for the development of the permanent teeth, and presenting at about the fifth month, the rudiments of a dental germ. Formed at the expense of the primitive dental sacs and by a new folding, a new *involution* of the mucus, the dental sacs of reserve are soon directed towards the posterior part of the alveolar border ; then as fast as the first teeth develop, they descend, and finally occupy a position behind and below the border. Each of them then presents, at its summit, a fibrous cord, which, according to Kolliker, prolongs itself for the incisors and canines to the buccal mucus; for the two small molars to the periosteum which invests the bottom of the two temporary molars. It is this fibrous cord which, improperly, has received from M. Serres, the name of *gubernaculum dentis ;* its function is yet unknown. As to the large molars of second dentition, their appearance takes place towards the last of the fourth month, by the development at the extremity of the primitive dental groove of a follicle intended for the formation of the first molar. This then leaves an interval between it and the gums where a little sac of reserve is formed, the development of which does not begin until towards the eighth month after birth, for the formation of the large molars. At length the wisdom tooth, the appearance of which, as is well known, is extremely variable, is formed by the reproduction of the same phenomena, from the posterior and superior part of the preceding tooth. *

### CHAPTER II.—*Structure of the Dental Follicle.*

In order to study the anatomical constitution of the dental follicle we have opened a great number of fœtal jaws of about from four to five months old, and we have been able, about this time, to observe the follicle immediately before

* See the plates in the Memoir of Goodsir (loc. cit.)

This evolution of the dental follicle, described with remarkable clearness by Prof. Goodsir, and adopted by the greater number of anatomists, had been already conjectured in France, by De Blainville, in 1822, in his theory of the development of the phanère; † "On a little reflection," says he, "the structure of the crypta and the phanère, considered abstractly, will appear to resemble the skin in structure; the fibrous capsule is the derm, the vascular net-work, corresponding to the membrane of this name; then comes a true pigment or colored matter, then the nervous system; even the epidermis has an analogy in the part produced from the crypta and from the phanère, so that one may form an idea of these organs by supposing the skin a depression or slight corrugation, which draws with it all the constituent parts."—(*Organization* des animaux, t. 1, p. 39.) If we can believe that the tooth, according to De Blainville, is a true phanère, we can comprehend how he applies to the dental papillæ the determination of phaneriferous substances in general. Be that as it may, Purkinje, Raschkow, M. M. Serres and Duvernoy, have maintained an opposite theory; these authors affirm that in the early fœtal life, the follicle is entirely free at the bottom of the jaw, without having any adherance to the gum, and consequently cannot be considered as dependent on it. We have, nevertheless, observed in a fœtus, less than three months old, that it was impossible to separate the gum from the subjacent follicles, and the surest means of obtaining these last, at such an early period, is to raise carefully the edge of the gum and look for them underneath. Moreover, the examination of the gum and the parietes of the follicle, shows a great similarity of structure, and the theory of Goodsir, adopted and defended by Kolliker, Tomes, Huxley, etc., seems then to approach the truth. Still the disagreement which exists on this question, appears to us to indicate the propriety of further research. It is incontestable that there is a close adherance, from about the third month, between the follicle and the gum, an adherance which, contrary to the opinion of M. Serres, will not permit heir separation without tearing.

† Arbrisseau grimpant qui croit dans la Cochinchine.—*Sup. au Dict. de l'Academie, Paris*, 824.

the beginning of dentification, that is to say, when the organs which it contains, arriving at their complete development, are prepared to perform the functions for which they are intended. Under these circumstances, this follicle appears to us to be composed of three parts, to wit:—

1st. The parietes or envelop of the follicle.

2d. The dental bulb, dental germ, or dental papillæ and the germ of the dentine or ivory.

3d. The germ or organ of the enamel.

The study of these parts induces us to divide this chapter into three paragraphs.

### SECTION I.—*Parietes or Envelop of the Dental Follicle.*

The parietes or envelop of the dental follicle, is constituted by a double sac placed at the bottom of the alveolar groove, and contains all the elements which go to form the teeth. (pl. 1, fig. 1, a, b.*) This sac is composed of two distinct membranes, which are easily separable at the beginning of dental production, but intimately united together when the crown of the tooth is formed. The nature of this envelop appears to us to present certain modifications which follow the different phases of the development of the dental organ. Thus before the beginning of dentification, it is composed only of a mass of cellular fibres, loosely united around the pulp which occupies the centre. At the moment when the germs of the enamel and the ivory have acquired the volume necessary to their functions and fill the cavity of the sac, the envelop can easily be separated into two membranes, which later become one, and contract an intimate adherance with the neck of the tooth, when the formation of the crown of the tooth is achieved. These two membranes, when separable, having each a particular structure, and seeming to play a

* The plates referred to in this translation will appear at the end of the work.

different part in the dental development, we should, therefore, study and describe separately. *

1. *External Envelop or Dental Capsule.*—The external envelop is a whitish and opaque membrane forming a complete sac, adhering, by one part, to the under face of the gum with the tissue of which it is intimately connected, and continuing by the other part, on the vascular and nervous pedicle which it traverses in order to reach the pulp. Its density, which is very considerable at the level of the gum, gradually diminishes until the envelop becomes very delicate at the opposite point. It is composed of a cellular net-work which resembles that of the mucous of which it seems to be a fold; a great number of capillaries interiorly ramify it and communicate directly with the vessels of the buccal mucus. It carpets by its external face, the walls of the alveoli, with which it is loosely united, and later, when the development of the tooth is complete, it extends over the root to form the alveolar periosteum or alveolar dental membrane.

2. *Internal Envelop.*—This membrane, like the preceding, represents a complete sac, of which the only opening, corresponding to the bottom of the alveolus, gives passage to the vessels and nerves of the pulp; it is thin, transparent and of a whitish color. Externally it is in contact with the preceding membrane from which it is easily separable until towards the period of birth, whilst later it is closely united to it and soon disappears as a distinct membrane. This union no doubt induced Blake and M. Oudet to believe that it became completely atrophied, and received from the first of these authors the name of *caduque* membrane. The internal face, smooth and polished, gives place, at two opposite points, to two germs of enamel and ivory. The first of

* According to Hunter, the dental follicle is surrounded by two concentric membranes, the external without vessels, the internal vascular; according to Blake, on the contrary, the external is vascular and the internal is without vessels. Meckel, E. Weber, Fox and M. Serres say that both are vascular membranes, after Dietrich, (Auleitung das alter der Pfende zu erkennen, p. 72, 1822;) later they both undergo ossification.

these organs adheres by its central part to the point corresponding to the insertion of the follicle to the gum, and receives by this means the capillaries which cross it. Whilst the germ of the ivory occupies the opposite point corresponding to the entrance of the dental vessels. But neither of these organs, nevertheless, is carpeted, as has been said, by a fold of the internal membrane which in reality passes underneath them, and besides, they have no other external limit than that of the amorphous matter, in the midst of which are deposited the elements which constitute them.* The remaining portion of the internal aspect of the internal envelop is carpeted by epithelium, the cells of which are spherical or become pavmentous by reciprocal pressure and contain one or more nuclei.

The totality of the cavity circumscribed by the internal membrane varies in its contents. Completely filled at first by the two germs of enamel and dentine, the atrophy of the first, joined to the gradual augmentation of the diameter of the sac, soon forms a vacancy (pl. 1, fig. 1, j.) which will be found filled with a reddish, viscous liquid, in which generally floats the cells of enamel in progress of development, some fragments of the germ of the enamel,† and flakes of epithelium, the cells of which becoming free, often assume the most strange and varied forms. This liquid, analyzed by Meissner,‡ contains a little albumin, phosphate, chlorate and sulphate of lime, in man, free lactic acid, and in the calf, a free alkali.

*According to Bichat (Anat. génér. p.114,) followed by Delabarre (Odontologie, p. 10,) and M. Oudet (Dictionary in 30 vols, t. x, p. 97), the internal lamina of the follicle is a true serous membrane which, after having carpeted the internal face of the sac, folds itself upon the dental pulp in order to furnish it with a proper envelop described later as the *membrana præformitiva,* and to which Purkinje, Raschkow and Huxley attribute equally a serous character.

† Huxley, Quarterly Jour. of Microscopical Science, April, 1853, and Goodsir, (loc. cit.) say that they met with, on the inside of this membrane, vascular villosities formed of true prolongations, presenting an appearance analogous to that of a portion of the injected intestinal mucous membrane; these villosities are unquestionably nothing else than the déb of the enamel organ, still adhering to the sac and floating in the liquid.

‡ See Meckel's Archiv., t. iii, p. 114.

The internal envelop of the dental follicle is composed of a cellular web traversed by a thick net-work of capillaries, about the period of the formation of the enamel, for after the accomplishment of this work, it changes its nature and disappears. Thus, while the existence of the external membrane is permanent, as it afterwards forms the periosteum of the root, the internal envelop has only a temporary existence subordinate to that of the germ of the enamel. When the functions of this have ceased, and when absorption is effected, the vascular communication, which united it to the internal membrane, disappears, and this organ, which seemed to have no other office than to furnish the materials of nutrition during the successive phases of the evolution of the enamel cells, is metamorphosed ; its vessels are obliterated and the remainder of its substance seems to be confounded with the tissue of the external membrane.

SEC. 2.—*Dental Bulb, Dental Germ, or Germ of the Dentine, and Pulp or Dental Papillæ.*

The germ of the dentine occupies the lowest part of the dental sac ; it is a soft pulpy organ, intimately attached to the internal membrane of the follicle with continuity of substance. Most authors have admitted, as we have seen, that the internal membrane reaching from the sac to the base of the bulb, does not pass under it and fold over to form an envelop for this organ, without holding it in its cavity after the manner of serous membranes; but we have often observed in man, and more easily in the calf, that, the dental bulb is not so well supplied with fibres as the membrane, which can be detached from the walls of the follicle but not without tearing the parts.

Before the fourth month of fœtal life the dental germ is as large as the head of a pin ; of a reddish color and already traversed by vessels. It is surrounded by the follicular walls which as yet does not present any membraneous disposition, and forms a kind of loose cellular atmosphere around the organ.

Its constitution at this period is very simple. It is a mass of finely granular amorphous matter containing a great quantity of fibro-plastic (*embryoplastic*, Robin) nuclei. These elements (pl. 1, fig. 2) are then ovoidal or spherical and present rarely the prolongations with which we will see them provided later; they offer to view at last the most perfect identity with the same elements so abundant in the fœtus during the first part of gestation.*

Towards the fourth month, the dental bulb begins to undergo important modifications; its form becomes modified and reproduces exactly that of the future tooth. It is thus that they become prismatic for the molars, conical for the canines, and shaped like the mouth of a flute for the incisors. At this time we observe in the nuclei, successive changes, in virtue of which these become the point of departure of the laminar fibres, (cellular fibres,) destined to constitute the web of the pulp. We intend to study carefully this phenomena, which has been unknown to most authors who have written on the development of the teeth, and which deserves to arrest our attention.

* We give the name of nuclei and embryoplastic cells to a kind of element presenting two usually coexistent varieties; the first and more common variety, is characterized by the form of its free nuclei, ovoidal, rarely spherical, often without nucleoli; the second by spheroidal or ovoidal cells, more or less irregular, having a nucleus like a free nucleus. These are the elements, which, with a little amorphous matter, constitute in themselves alone, during a certain period, the tissue of the body of the embryo, (whence the word *embryoplastic*,) a grayish or whitish tissue, soft, friable, pulpy, semi-transparent and gelatinous, and called by the anatomist, cellular or mucous primordial embryonic tissue. Little by little the laminated fibres, the muscular fibres, develop in the embryoplastic tissue, which is finally replaced. The embryoplastic nuclei do not completely disappear, but remain during life as accessory elements to the laminated, fibrous, muscular tissues, etc.; from the first they have the names of nuclei, cells or globules and corpuscles of the cellular tissues, of globules or nuclei, and ovoidal fibro-plastoid cells, etc. It frequently happens that the embryoplastic nuclei, which predominate in the embryo and become accessories of the tissues in the adult, are affected in the adult with hypergenesis; they afterwards cause recurrent tumors, which possess, externally, tissue resembling the tissue of the embryo.

The embryoplastic nuclei are oval, rarely spherical, with well defined or slightly denticulated borders, in the normal, laminated tissue, where they are a little longer and less regular than in most other parts of the economy. Their normal length varies from 0mm, 007 to 0mm, 010, and their thickness from 0mm, 005 to 0mm, 006. They are insoluble in acetic acid; all of them contain fine molecular granulations and one or two nucleoli, distinct and brilliant. (Robin.)

On two opposite points of the nucleus, a well defined, but pale and delicate prolongation appears, its form is that of a cone, of which the base corresponds to the nucleus, and the fine extremity of which takes a rectilinear direction, if the amorphous matter which surrounds it is abundant, and the nuclei rare, and which on the contrary, takes a crooked, irregular direction if the nuclei are pressed against each other. The nucleus included in this manner between two prolongations becomes fusiform, (spindle shaped fibro-plastic bodies;) only it is necessary to observe, that it is not at the expense of its substance that the prolongations are formed, for these are formed around the nucleus as the centre of generation, as a condition of formation, (pl. 1, fig. 4.)

Soon after the birth of the two prolongations which we have just described, there are produced new prolongations on the different points of the nucleus, and these are soon surrounded by numerous rays, (stellate fibro-plastic bodies,) which thus forms the cellular net-work of the pulp, (pl. 11, fig. 1.*) When the laminar evolution of the embryonic nucleus is complete, they are absorbed and disappear at the same time that new nuclei come into existence in the midst of the organ, in order to undergo, in their turn, evolution.

The formation of the cellular net-work of the pulp, which we will examine under an entirely new aspect, is already far advanced towards the fifth month of fœtal life, and the constitution of the organ seems complete. It then is as large as a pea, and, physically, its characteristics are visible to the naked eye. It is a soft, reddish mass, completely unprovided with a distinct and separate membrane or envelop. Authors have nevertheless admitted the ex-

* Purkinje and Raschkow seem to have observed these last phenomena, when they speak of "angular granules united by very delicate threads of cellular tissue." M. Duvernoy describes them with still greater precision: "There can be seen," says he, "in thin sections of the dental bulb, periform, oval, angular, round bodies, having threads or tubes, which start out in rays from their pointed parts when their contour presents them, and which are joined by these threads to others of these bodies, (Dents des musaraignes, p. 85; 1844.) Kolliker, Lent and Haunover refer to the same thing when they speak of stellate cells of the pulp, (Kolliker, Histologie Humaine, p. 199.)

istence of a membrane around the dental germ, underneath which is formed the dentine and according to some the enamel itself.* It is necessary to understand thoroughly the precise nature of this membrane. There is not, in fact around the pulp a membrane visible to the naked eye and separable from the subjacent tissues by means of the scalpel, only the most superficial covering of the amorphous matter which enters into the composition of the germ, present a little greater density. But what it is necessary to well comprehend is, that there is an absolute continuity of substance between this superficial covering and the subjacent amorphous matter. Prolonged maceration sometimes brings about a separation of the two parts and it is then possible to see under the microscope the supposed membrane detached from the remainder of the organ and floating in the liquid in very delicate membraniform fragments; the result only of the difference of the density of the two parts and of their unequal resistence from the mode of preparation. As to the office it has to play in the formation of the dental substance, we believe it has none. We think, with all authors, that the cells of ivory are formed under them; but we believe that they disappear by absorption under the first formed layers of dentine. We cannot then admit with Huxley that all the dental substances, ivory, enamel and cement, are formed between this membrane and the germ which it covers, since the germ of enamel, the organ produced from this tissue, is entirely foreign to that of the dentine above which it is placed, as a bonnet, without adhering to it. Nor can we accept the opinion of Tomes who thinks that the cells of enamel traverse this membrane in order to place themselves on its lower face in contact with ivory formed. It was this supposed intervention in the development of the tooth which caused so much embarrassment to Kolliker and to Lent and induced them to offer two hypotheses, equally inadmissible, on the development

* This membrane has received different names: *membrane præformativa* (Raschkow,) *basement membrane* (Bowman, Huxley,) *persistent capsule* (Nasmyth.)

of the teeth, to wit: that the fibres of this tissue result, 1st, either from a secretion of the cells of the adamantine membrane, a secretion which traverses the *membrana præformitiva* in a liquid state and afterwards solidifies and ossifies: 2nd, that the fibres of enamel result from exuded plasma through the dental canaliculi.*

The dental pulp receives vessels and nerves. The vessels are extremely numerous, above all towards the beginning of dentification, that is to say from the fifteenth or sixteenth week of intrauterine life. They proceed from the pedicle of the pulp and divide into a great number of very delicate capillaries which terminate towards the middle of the organ without arriving at its surface. The meshes are extremely close and constitute a rich vascular net-work. The nerves, equally numerous, develop much later than the vessels. Their mode of termination, as yet little studied, seems to be made according to M. Robin, by free extremities and not by anastomosis like the capillaries. This extremity without sensitive or pacinian corpuscles, is conical or slightly swelled like a button.

About the time of the formation of the dentine the bulb becomes the seat of interesting particulars which have not yet been signalised. A small quantity of calcareous matter is observed to be produced in the substance of the organ, spheroid in form and having a volume of 0mm, 05, of diameter. These little spheres are very brilliant and possess an index of refraction approaching that of drops of oil. Their number is very considerable at the time of birth, that is to say, when dentification is actively commenced. They are insoluble in alcohol, ether, and carbonate of sulphur, but hydrochloric acid, without completely dissolving, renders them pale and granular, a chemical reaction which joined to the physical characteristics they present, sufficiently demonstrate that they are composed of phosphate of lime already combined with azotized matter which opposes itself to their complete dissolution in hydrochloric acid.

* Kolliker, loc. cit. p. 432.

This curious production of calcareous matter, which we have recognized in ruminants and rodents, ceases when the tooth has acquired its full development, so that up to this time the dentine continues to produce it on the parietes of the dental cavity. It seems then very logical to conclude that this production is due to an exaggeration of the organic movement, of which the pulp becomes the seat during the early period of dentification, to a considerable afflux of calcareous matter of which a part, beyond the requirements of dental formation, is deposited in the thickness of the germ under form of an amorphous mass. This explanation seems the more probable as conjoined to the existence of the calcarious matter, we meet it in the germ of the depots of hematoidin either amorphous or infiltrated, or crystalised in tufts or needles, (pl. 1, fig. 3, E, E.) This new circumstance is explained also by an exaggeration of the organic movement and a very considerable sanguine afflux. These two phenomena are often presented to our observation and seem to us if not constant at least very frequent in the period when dentification is in full force. (For the remainder of this description see explanation to the fig 5, pl. 1.)

### SECTION 3.—*Germ of the Enamel.*

Bulb or organ of enamel, (Kolliker,) *organon adamantinæ*, (Purkinje.)

If we open with care the dental sac at the moment when dentification has commenced or just before, we will observe a great quantity of gelatinous matter adhering to the internal surface of the walls of the sac and covering the germ of the dentine. This mass, transparent, and sticky to the touch, is the germ of the enamel. Its form varies with the teeth it is called upon to cover; it has also the appearance of a bonnet for the incisors and canines, (pl. 1, fig. 1, c.,) but for the molars, it affects the form of a double convex lens, and is lodged in the concavity of the pulp, and is found as entwined by it; until the ivory is developed, the tubercles of the crown are forced into the organ, which then expands to cover the whole surface of the formed ivory.* Its size about equivalent to the third or to half the size of the germ of the dentine, and its preparation, under the microscope, often presents great difficulty, on account of its extreme friability and of the impossibility which one experiences in trying to lacerate it by means of the needles. Its gelatinous consistence will always, at least, permit it to be placed between two bits of glass to be observed thus without other preparation †

This organ is connected by its external or follicular face, with the internal membrane of the sac, to which it adheres and which it furnished with its vessels; the other part corresponds by its internal or dentinary face to the projecting part of the bulb of the ivory upon which it moulds itself, and of which it follows the outline and sinuosities. As to its free edge, it descends more or less on the sides of the dental bulb, without, however, reaching to its base.

If we now observe the parts constituting the follicle together, (pl. fig. 1,) we can state that its cavity is found nearly filled by two organs inserted on two opposite points of the wall. The first of these organs, fixed at the bottom of the sac, is the germ of the dentine, (pl. fig. 1, i;) the second inserted on the point directly opposite, is the germ of the enamel, (pl. 1, fig. 1, c.) These two organs thus direct themselves towards the centre of the follicular cavity, they meet and fold one upon the other, until the production of the ivory and the enamel, which operates between them,

* It is these organs, which with ruminants and pachyderms, affect a membranous disposition, in order to force themselves into the divisions of the crown, and to cover the enamel. With rodents, it occupies the anterior face of the base of the incisors, and by a similar process produces the enamel as fast as the bulb produces the dentine. The result is, that with these animals, the incisors are only covered with enamel on their anterior face.

† Its consistenee is so soft, that putrefaction rapidly carries off its liquid. On pieces a little old, one may recognize the presence of this organ, which does not appear under the water but with the aspect of a cloud floating in the liquid.

separates and removes them one from the other, and later determines their complete atrophy of the organ of the enamel, of which the functions are temporary ; incomplete for the organ of the dentine, the function of which continues through life, and, whilst the summit of the germ of the dentine covers the cells of ivory, (pl. 1, fig. 1, h,) the corresponding face of the germ of the enamel has charge of other cells, cells of the enamel, (pl. 1, fig. 1, e,) which glue themselves to the first laminæ of ivory formed, and are transformed into columns or prisms of enamel.

The germ of the enamel is composed then, according to our view, of two distinct parts, (pl. 11, fig. 1,) but intimately united together, and bound together by their functions: 1st, the gelatinous mass ; 2d, the cells of the enamel, which we shall study before long, (see Development of the Enamel.) We see then, that for a great number of authors, the germ of the enamel is reduced to a membraniform series of cells, (adamantine membrane.) Hannover* even goes further, and if we may believe him, the gelatinous part would represent the germ of the cement, and the cells the germ of the enamel, whilst one particular membrane (intermediate membrane,) would separate these two parts from each other.

The structure of the organ of enamel, much resembles that of the dental pulp ; thus we find it formed of a mass of amorphous matter, very pale and very transparent, (pl. 11, fig. 1, a,) in which is placed a quantity of embryoplastic nuclei, of which we are able to follow the different phases of evolution up to the moment when they have produced a complete cellular web. This phenomenon is much more well defined and much more complete than in the germ of the ivory ; the nuclei are a little more voluminous and less pressed together, the prolongations more numerous and com-

* Uber die Entwickelung und den Bau des Säguethierzahns, (Nova acta Acad. Cœsari Leopoldi nat, cur., vol. xxv, part 11, p. 819.)

plex, (pl. 11, fig. 1, c,) and the web which results of remarkable elegance.*

The dentinal face of the enamel organ offers, as we have said, a continuous range of cells, to which the authors have attributed a membranous tendency, (adamantine membrane, Raschkow,) and which Kolliker, (loc. cit. p. 426,) regards as an epithelial bed. These cells, which form part of the enamel organ, are no other than those which, in consequence of successive transformation, are called upon to form the enamel of the crown ; so that not only do the two great productive organs of the tooth present great analogy of anatomical constitution, but also do the modes of formation of enamel and ivory obey the same law ; the production and successive transformation of the special cells, we shall develop in the last part of this last proposition.

Contrary to the assertions of Owen, Nasmyth and of Todd and Bowman,† the enamel germ is provided with a net-work of capillaries less rich, it is true, than that of the dental bulb, and which proceeds from the vessels of the internal membrane of the follicle; but our observations have not succeeded up to the present time, in proving in this organ, the presence of nervous fibres, and authors, moreover, have not made any mention of them.

### PART SECOND.

### *Development of the Teeth.*

### CHAPTER I.—*Development of the Dentine.*

#### A. FACTS.

In order to observe with accuracy, the formation of the ivory, it is necessary to open a dental follicle, in which the germ is yet only covered with an extremely thin dentine;

* This appearance Kolliker described under the name of stelliferous cells of the organ of enamel, (Histologie Humaine, p. 431,) and which has been observed by Todd and Bowman, (Physiological Anatomy, 8vo., vol. 11, p. 175; Lond., 1847.)

† See Owen, *Odontography*, Introduction, p. lix.—Nasmyth, Researches on the Development, Structure and Diseases of the Teeth, p. 109, 1839.—Todd and Bowman, loc. cit. 11, p. 175.

one should then break this covering with precaution, and do it in such a manner as to draw with one of the fragments, the correspondiug part of the pulp.* One will thus be able to see the whole preparation on the field of the microscope, at a glance: on one side, the ivory completely developed, on the other, the cells of the ivory, and all the intermediate degrees of development between these two extreme points. The cells of dentine observed under these circumstances, present themselves under the aspect of regular, superimposed rows, of which the direction follows the contour of the pulp and situated in the midst of the amorphous matter which surrounds this organ (pl. 1, fig. 1, h, and fig. 5, c c.) Thus united and placed side by side they assume a cylindrical or prismatic form caused by reciprocal pressure; it is then quite difficult to study their characteristics and their nucleus is rarely visible.

Observed separately (pl. 1, fig. 7,) that is to say, detached from the preceding preparation or taken from the surface of the pulp immediately before the commencement of the formation of ivory, the cells of dentine have variable forms; they are spherical, ovoidal, or periform ; their length varies from 0mm, 02 to 0mm, 04 ; their thickness from 0mm, 003 to 0mm, 015. Their outline is extremely faint; their contents granular, but equally pale. The nucleus which they nearly all contain is very deep colored, very large relatively to the cell ; it is ovoid or spherical and its diameter sometimes attains 0mm, 01, so that it then occupies the entire width of the cell of which it even passes the walls. It is sometimes found half contained in the body of the cell whilst the other half juts outside. The peripheral extremity, which corresponds to the nucleus, enlarges some-

* This preparation, is often very difficult to realize on account of the weak adherance of the layers of dentine with the subjacent pulp ; it was only after numerous fruitless efforts that I was able to obtain a true demonstrative, human preparation. This is represented by pl. 1, fig. 5, which also answers to my description. With ruminants the preparation is easier, and I possess in my collection, several pieces taken from the calf, and which show very clearly, the cellular disposition of the newly formed dentine.

times to receive it, whilst the opposite extremity tapers into a point and is sometimes terminated by a kind of long cue, sinuous and very pale, for the granulations assemble in the nuclear part of the cell. This point, the thickness of which often attains to double the size of its cell, is not constant; it is ordinarily single, rarely bifid (pl. 1, fig. 7.) Answering always to the surface of the pulp, in the amorphous matter of which it is spread more or less freely, it is absorbed at the beginning of calcification, and we find no further trace of it. This cue is found in the dental cells of all animals. It is rare in rodents; but in ruminants and the horse we have seen it very frequently and gifted with a great development.

The nucleus is formed of deep colored granulations, among which we find some very brilliant; its outline is so dark that it is often barely visible, so that we can hardly perceive the extremely pale limit of the cell. This seems very fragile for we have found it more or less broken, whilst the nucleus which is resistant, subsist and finds itself isolated. This nucleus seems to us to exist before the formation of the cell around which the elements group themselves. What seems to prove it is, that in the superficial coverings of the dental pulp we find a great number without the cell and it is impossible, in this case, to suppose that they have been broken or destroyed.

Chemical reactions have a curious effect on the cells of the dentine. Alcohol and the acids affects similarly to other anatomical elements, but glycerine renders the nucleus pale and completely dissolves it in some hours without attacking the cell; we have seen the enamel cell, on the contrary, under the same circumstances, present the inverse.

The first row of cells which appear in the thickness of the amorphous matter which surrounds the bulb, in rising, lifts up the more superficial layer, which atrophies at a later period. At this moment, each cell still presents the characteristics we have assigned them; but we are soon able to see changes produced of which the beginning is marked by a disappearance of the nucleus, which is entirely absorbed. The cells press against each other, elongate, (pl. 1, fig. 5, c,) become prismatic and commence to undergo the phenomenon of calcification, which makes the soft cell, a little hard mass of dentine, (pl. 1, fig. 6, b.) The granulations then disappear, the row of cells simultaneously transformed, present an homogeneous aspect, (pl. 1, fig. 6, a,) and before dentification is achieved, a new row of cells are produced under the first, and it, in its turn, undergoes the same modifications. We thus see that ivory is formed by beds of superimposed cells which harden separately, and it is not astonishing that afterwards, in an adult tooth, they decompose this in a number of little concentrate layers.

### 1*st. Formation of the Canaliculi.*

A great number of hypotheses have been offered in science, upon the mode of the formation of the canaliculi ; we are, nevertheless, able to reduce them to two leading opinions, which we will study successively.

1st Opinion. The first opinion considers the canaliculi as a dependance of the cell ; it is defended by Kolliker, Lent, Hannover, Tomes, etc. According to the two first authors,* each cell of the dentine produces, by its non-nuclear peripheral extremity, a tubular prolongation, which elongates considerably, divides into numerous ramifications, anastomoses with the ramifications of the neighboring cells, and thus gives rise to the complicated net-work of the tubes of the ivory. We have shown, in effect, that a great number of isolated cells of the ivory, present prolongations very rarely bifid ; but these have never seemed hollow to us, and we judge that they are directed constantly from the side of the pulp, that is to say, inversely to the direction of the canaliculi. Hannover † professes a doctrine slightly differ-

* Kolliker, Histologie Humaine, p. 432, f. 200 and 201.—Lent, Beiträge zur Entwickel d. Zahnbeines und Schmelzes, in Zeitchr. f. wiss, Zool., vi, 1 ch.

† Loc. cit., p. 810.

ent, considering the canaliculi as composed of a distinct wall of the cavity; he admits that the nucleus of the cell elongates, forms the cavity (*lumen,*) of the canaliculi, and that the cell forms the wall of it. According to Tomes,* whose opinion approaches that of the preceding author, the cylindrical cells of the dentine unite from one end to the other; the nuclei are nothing else, according to him, and like Hannover, than a cavity made in the thickness of the cell, elongated, following its axes and reuniting the one to the other, and thus constituting the canaliculi, whilst the cell becomes the intertubular tissue.

We would object to these two last explanations, as the nucleus cannot be regarded as a cavity made in the mass of the cell, since it is frequently met with isolated and broken into several fragments, and which, besides, far from extending in dimensions, rapidly absorbs and disappears.

2d Opinion. The second opinion, defended by Schwann, Raschkow and Huxley,† considers the canaliculi as constituted by the intervals left between the dentinal cells, intervals which correspond in all the rows of cells, and thus forms a tube which traverses the entire thickness of the tooth. This opinion seems to us to conform to observation. If one examines, indeed, under the microscope, the lower face of a cap of dentine in progress of development, (pl. 1, fig. 6,) it is shown that the outline of the base of the cells is formed by a whitish and transparent line. If the focus of the microscope is varied, the direction of this line is easily followed, and is observed to join, in most cases, to a white, circular and transparent point, which is nothing else than the orifice of the canaliculi, open to the surface of the pulp, (pl. 1, fig. 6, b,) and in other cases it continues with a canaliculus to the middle of the ivory, in the interval of two calcified

* A Course of Lectures on Dental Physiology and Surgery, p. 86, 1848.

† See Schwann, Mikroscopische Untersuchungen ueber die ueberenstinnung in der Structur und den Wastrothum der Thiere und Pflanzen; Berlin, 1839.—Raschkow, Meletemata circa dentium mammalium evolutionem; Breslau, 1835.—Huxley, Quarterly Jour. of Med. Science, April, 1853.

cells, (pl. 1, fig. 6, a.) These whitish lines, circumscribing the periphery of the cell, plainly present a grooved aspect, which the sticking of the new cells should convert into a complete canal. The distance which separates the canaliculi between them is then measured by the thickness of the cell. This distance is, however, very variable, since the diameter of the cell is not constant. This circumstance is owing to the fact that each little mass of dentine undergoes, during its development, organic modifications, in virtue of which they elongate more or less, so that the canaliculi approach each other. It is this which takes place by the formation of the layers of the dentinal cells, when the cavity of the pulp has acquired the adult dimensions. As to the anastomoses, they are produced in part by the furrows which surround, primitively, the base of the cell ; in part by the molecular reabsorption affected at the bottom of the organ, in consequence of the organic movement of formation and decomposition of which this tissue is the seat. We will hereafter dilate on these considerations.

### 2d. *Globules of Dentine.*

When we examine the lower face of a cap of dentine in process of evolution, magnified two or three hundred diameters, we meet with, nearly always, a more or less considerable number of little mamelons, of different sizes, and making an integral part of the dental tissue. These little masses are the *globules* of dentine, (*tooth substance balls* of the English,) described lately by Czermak,* and Salter,† These are spherical or ovoidal bodies, possessed of great transparency and limited by distinct and dark outline, (pl. 11, fig. 7, a.) They are composed of normal dentine, and are traversed in the ordinary manner by tubes. Their dimensions vary from the smallest size to a size appreciable to the naked eye. Their presence is constant in all the teeth, but their number is very varia-

* Zeitschrift fur wessenschaftliche Zoologie of Seibold and Kolliker, t. 11, 1850.
† Quarterly Journal of Microscopical Sciences, July, 1853.

ble. Sometimes the ivory is composed exclusively of them, sometimes they are to be found in it here and there. In certain cases they are so very numerous, that they form by their aggregation, masses which are discoverable on the wall of the dental cavity, by the naked eye, where this phenomenon is ordinarily met with.

Formed independently, these globular masses intercept, by their contact, the triangular spaces, with curved surfaces, (pl. 11, fig. 5, d,) which by false analogy of form, joined to the ignorance of the mode of their production, have caused them to be taken for modified osseous corpuscles. These spaces are ordinarily hollow and filled with air, which gives them, under the microscope, a black color ; and, like the globules, they exercise no influence upon the direction and progress of the canaliculi, which conjoin in their interior, as they join elsewhere, without one can see in this case, a special tendency in support of the approach of which we have been speaking. Kolliker believes, that under certain circumstances, these interspaces contain a peculiar soft substance, which the canaliculi pass through ; but Salter denies this. Be that as it may, their great number, on the ivory of a tooth, should constitute a vice of formation, of which we should be able to foresee the early evil influence, in the progress of certain dental affections ; caries for example.

The production of the globules of ivory seems to us, due to an irregular accumulation of calcareous matter in the midst of the dental cells, a kind of new exaggeration of the organic movement, an exaggeration of which the pulp has already given us proof by the presence of hématoidin crystals and phosphatic masses. When a row of dental cells has been produced, the afflux of calcareous matter remaining, is considerable ; it forms on the interior row, a species of vegetation, which, operating in contact with a soft and depressible organ, the dental pulp, they take a spherical form. Endowed originally with a very minute volume, these eminences of dentine, as one may call them, grow with the arrival of new substances, until a new layer of ivory, adding itself to the mass already formed, stops their development. We think, then, contrary to the opinion of Czermak, that globular masses of ivory are solid bodies, composed of normal tissue, and traversed, according to their size, by a more or less considerable number of dental canaliculi, of which the direction does not undergo any modification, (pl, 11, fig. 7, b.) It is necessary to add, that with the progress of age, and the molecular changes of which the ivory is the seat, these globular masses ultimately unite, whilst the interglobular spaces merge together and gradually disappear ; it is also difficult, on the section of the tooth of an old man, to prove, by transmitted light, the circular lines and the dark triangular spots which are so easily seen in the teeth of adults or young persons, and which indicate the outline of the globular masses and the lacunes which they intercept.

The explanation which we have here given of the mode of formation of these globules, approaches that of Czermak and Salter, who compare them to stalactites, produced at a moment, when the development of the ivory has ceased, an event which we believe due, in most cases, to a general cause which will have its counter effect in the development of the enamel, where it will produce transverse grooves, these enfractuosities often so deep, that in meeting with them upon the crown of the teeth said to be eroded, and which practitioners have remarked, for a long time, as almost always the result of an affection of infancy, which modifies or momentarily stops the work of dentification.

[To be continued.]

