# Errata

**Published:** 1859-10

**Authors:** 


### ERRATA.

The Translator of Magitot "On the Development of the Human Teeth," greatly regrets, that, through negligence in proof reading, many gallicisms and worse mistakes require corrections. He begs the indulgence of the reader in offering the by no means exhaustive errata which follow:

For *Docteun*, in the heading of the Article, p. 153, read *Docteur*.

Page 154, line 6 from top, for *Odontolgeny*, read *Odontogeny*.

155, " 14 " " for *complete* read *completed*.

157, " 11 " " insert *gingival* after *the*, and strike out the words *connected with the gums*.

157, line 28 from top, insert a comma after *molars*.

157, " 29 " " insert the words *of the alveoli*, after *bottom*.

157, " 34 " " insert a comma after *development*, and one after *groove*.

161, line 2 from top, read *in the gum*, for *to the gum*.

161, " 21 " " for *floats*, read *float*.

161, note at bottom of page; for *déb*, read *débris*.

162, " 3 from the bottom, for *does*, read *do*.

162, " 2 " " for *forms* read *form*.

163, " 6 " top, for *at last*, read *indeed*.

163, " 18 " " for *phenomena*, read *phenomenon*.

164, " 6 " " for *and*, read *but*.

164, " 18 " " for *forms*, read *form*.

164, " 19 " " for *nucléus*, read *nuclei*.

165, " 21 " " for *them*, read *it*.

166, " 30 " " for *carbonate of sulphur*, read *sulphide of carbon*.

167, line 10 " " insert *the*, after *under*.

168, " 4 " " insert *is*, after *size*.

169, " 2 " " for *of the organ*, read *for the organ*.

169, " 5 " " insert a semicolon after *life*.

174, " 20 " " for *forms*, read *form*.

159, " 6 " " for *envelop*, read *envelope*; and elsewhere in the Article, read envelope.

